# Supplementary material for: Persistent flat band splitting and strong selective band renormalization in a kagome magnet thin film
Source: Nat Commun. 2024 Oct 30;15:9376. doi: 10.1038/s41467-024-53722-3 (PMC11526111; doi:10.1038/s41467-024-53722-3)
Supplement: Supplementary file 1 — Supplementary Information [file 41467_2024_53722_MOESM1_ESM.pdf]

Supplementary information of "Persistent flat band splitting and strong selective band renormalization in a kagome magnet thin film"

Supplementary note 1 Topology of the flat band and the compact molecular orbitals

While the compact localized states capture the destructive interference nature of the kagome flat band, they do not form an orthogonal and complete basis [1]. Furthermore, due to the topological nature of the kagome flat band, exponentially localized Wannier orbitals for the flat band alone are prohibited [2]. Indeed, our DFT calculation in the PM phase shows that the flat band at E_F is topologically non-trivial (Supplementary Figure 1). Therefore, strictly speaking the local magnetic moments are not due to the conventional compact localized states. Nonetheless, they motivated the construction through a proper Wannerization of the kagome flat band and other dispersive bands, which results in the compact molecular orbitals (that coexist with extended molecular orbitals) [3]. Such compact molecular orbitals form a complete and orthogonal basis, and also overcome the topological obstruction. A Hubbard model defined with this basis produces a phase diagram as shown in Ref. [3], where a moderate Hubbard U in the new correlation regime ($D_{flat} < U < D_{wide}$) [4] gives rise to a local moment magnetic order associated with the compact molecular orbitals (Fig. 1j), consistent with the experimental observations here.

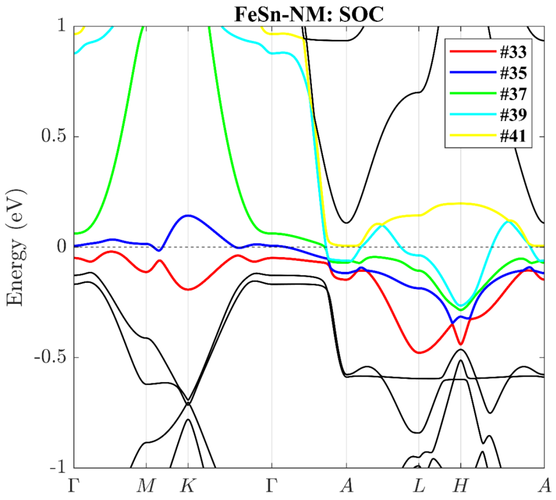

Band index	Parity				Product of parities				Z_2
	Γ	M	A	L	Γ	M	A	L	
41	1	1	1	1	1	1	1	1	1
39	1	-1	1	-1	1	1	1	1	1
37	1	1	1	-1	1	-1	1	-1	1
35	1	-1	1	1	1	-1	1	1	-1
33	1	-1	1	-1	1	1	1	1	1

Supplementary Figure 1 Calculated band topology of the bands near E_F in the PM phase.

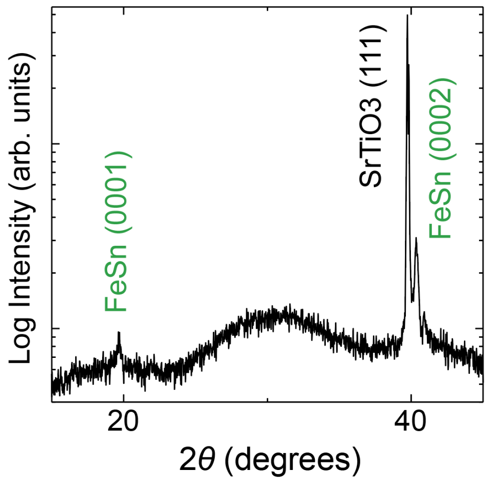

Supplementary Figure 2 X-ray diffraction spectra of FeSn/$SrTiO_3$(111). FeSn and $SrTiO_3$ peaks are labeled in green and black, respectively.

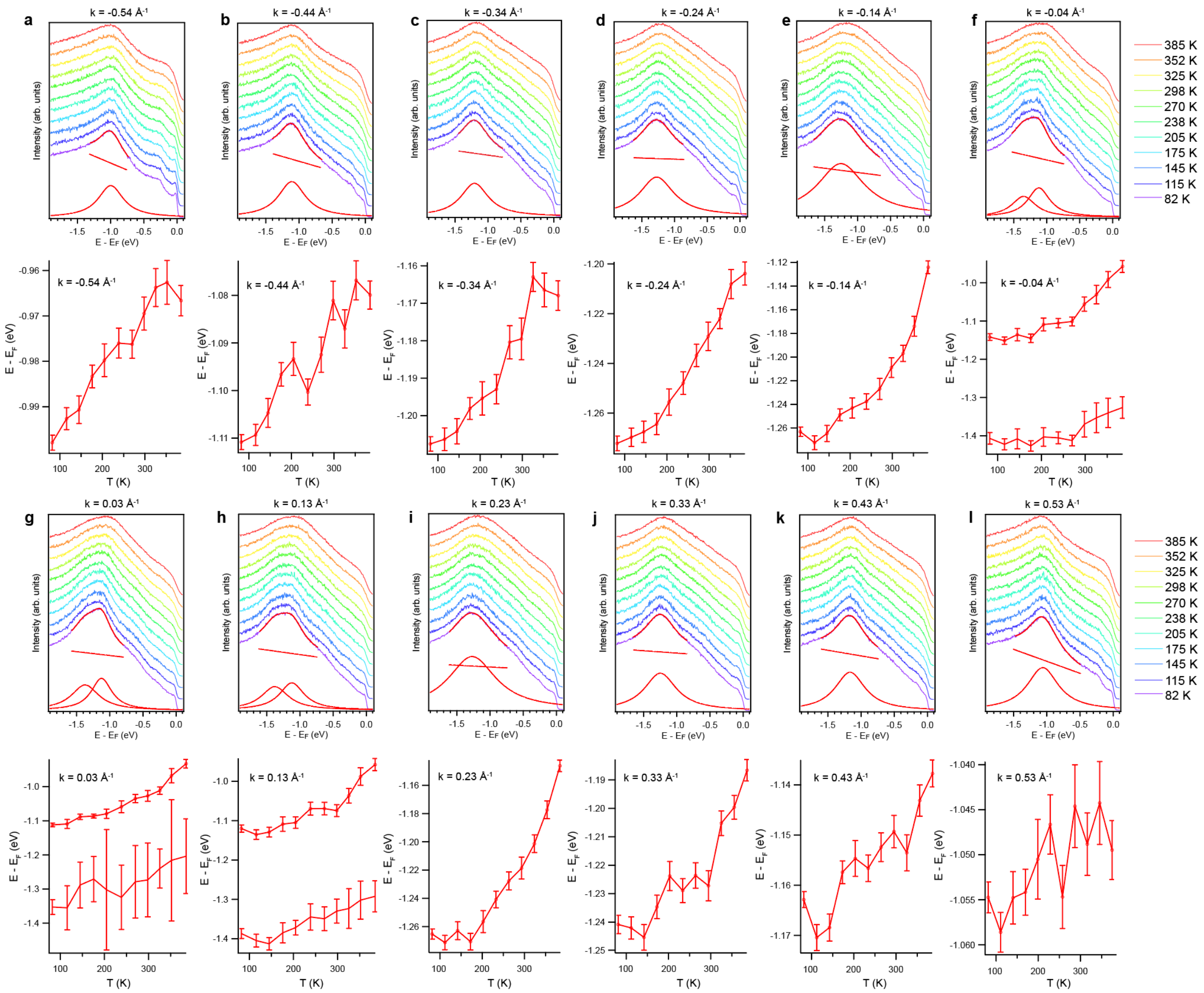

Supplementary Figure 3 Fitting details of the EDCs from the $\overline{\mathrm{K}}-\overline{\Gamma}-\overline{\mathrm{K}}$ cuts associated with Fig. 2d-i. a-**l** Temperature-dependent EDCs and their fitting results, taken at *k* ranging from -0.54 Å^{-1} to 0.53 Å^{-1}, respectively. In each panel, upper row shows the EDC stacking as a function of temperature extracted from the $\overline{\mathrm{K}}-\overline{\Gamma}-\overline{\mathrm{K}}$ cuts at the momentum labeled at the top of each panel. We fit each EDC within an energy window across the peak using the sum of a linear background and a Lorentzian function (or two Lorentzian functions for **f**-**h**). The fitted curve, linear background within the fitting range and the Lorentzian function are plotted in each panel in red color for the 82 K EDC. Bottom row of each panel shows the peak position of the Lorentzian functions as a function of temperature. Error bars are from the standard deviation resulting from the fitting process.

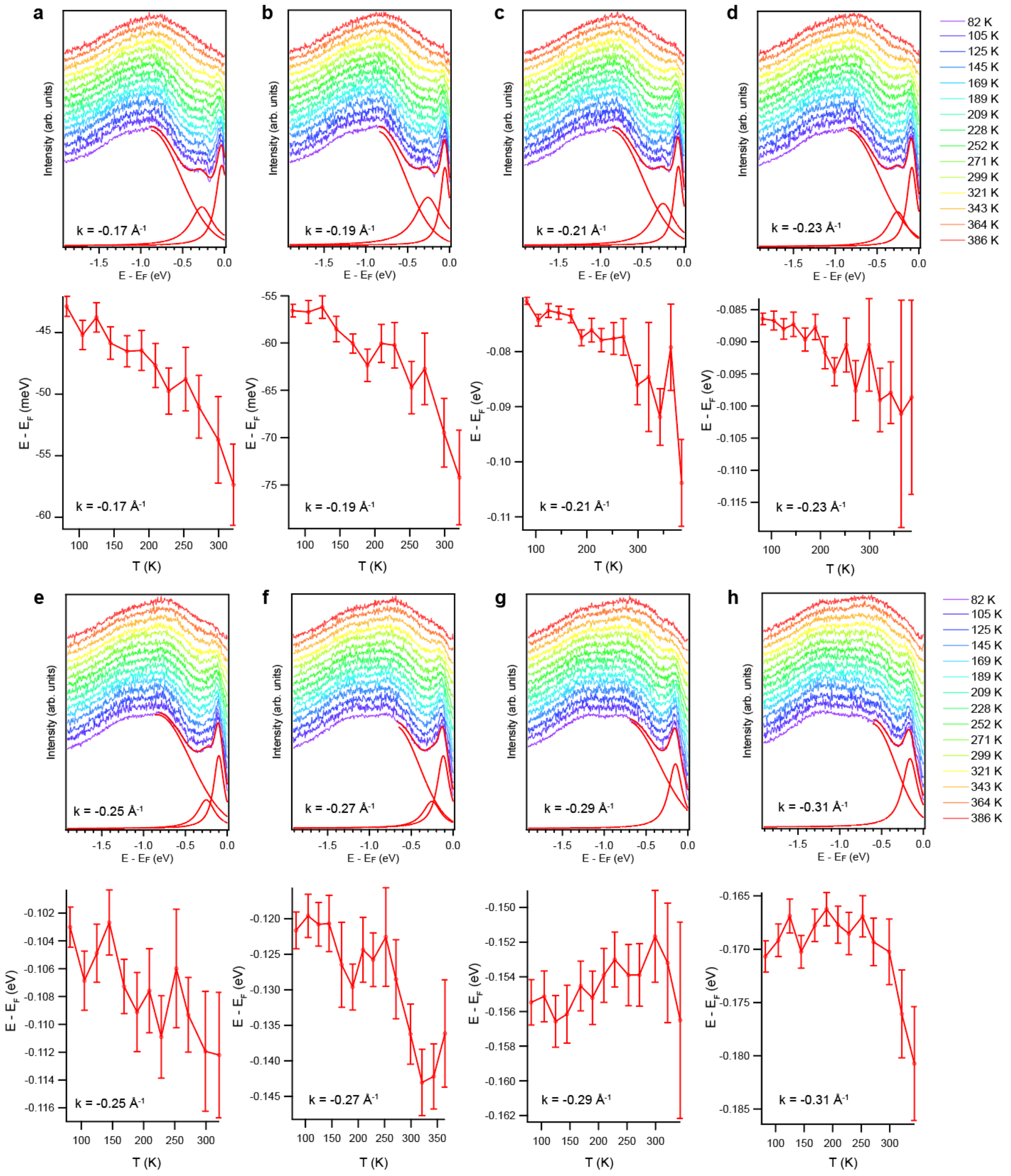

Supplementary Figure 4 Fitting details of the EDCs from the $\overline{\mathrm{K}}-\overline{\mathrm{M}}-\overline{\mathrm{K}}$ cuts associated with Fig. 2j-o. a-h Temperature-dependent EDCs and their fitting results, taken at *k* ranging from -0.17 $Å^{-1}$ to -0.31 $Å^{-1}$, respectively. In each panel, upper row shows the EDC stacking, after the Fermi-Dirac function is divided, as a function of temperature extracted from the $\overline{\mathrm{K}}-\overline{\mathrm{M}}-\overline{\mathrm{K}}$ cuts at the momentum labeled in each panel. We fit each EDC within an energy window across the peak using the sum of a Gaussian background and two Lorentzian functions (or one Lorentzian function for **g**,**h**). The fitted curve, Gaussian background within the fitting range and the Lorentzian functions are plotted in each panel in red color for the 82 K EDC. Bottom row of each panel shows the peak position of the Lorentzian function closer to E_F, which corresponds to the band in the Dirac cone, as a function of temperature. Error bars are from the standard deviation resulting from the fitting process.

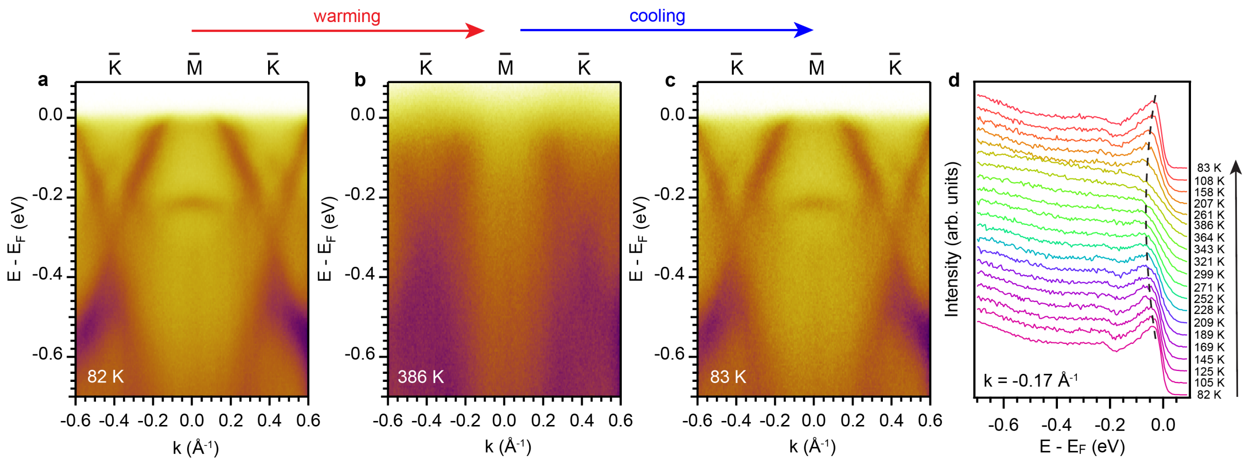

Supplementary Figure 5 Thermal cycling results of the the $\bar{\mathbf{K}}-\bar{\mathbf{M}}-\bar{\mathbf{K}}$ cut. a Initial cut taken at 82 K. **b** Cut taken at 386 K, warmed up from 82 K. **c** Final cut taken at 83 K, cooled down from 386 K. **d** EDCs taken at k=-0.17 Å^{-1} as a function of temperature, in chronological order as indicated by the arrow direction. Blacked dashed line tracks the EDC peak corresponding to the Dirac cone near E_F and shows the recovering of the same band structure after thermal cycling.

Supplementary Note 2 Impact of lattice thermal expansion on band shifts

To exclude the effect of thermal lattice expansion as a potential origin of the observed band shift, we carried out additional DFT calculations to estimate the magnitude of the effect of lattice expansion. Regarding band shifts as a result of lattice change with temperature, a previous study found an in-plane lattice expansion of 0.4% over a 300 K temperature window [5]. To explore the change of band structure due to the change of lattice parameters, we performed DFT calculations as a function of strain. Supplementary Figure 6 shows the comparison of the DFT bands with in-plane zero (blue) and 0.5% tensile strain (red). Although discernible changes exist, most of the bands shift towards lower binding energy with tensile strain. In particular, the band bottom at Γ and the top of the Dirac bands (green boxes in Supplementary Figure 6) shift in the same direction, contradicting the temperature-dependent ARPES results. Furthermore, the predicted shift at the band bottom near Γ with 0.5% tensile strain is ~0.04 eV, whereas the ARPES data shows a ~0.2 eV shift from 82 K to 385 K. Therefore, band shifts induced by lattice expansion cannot account for the direction nor total amount of the shifts observed.

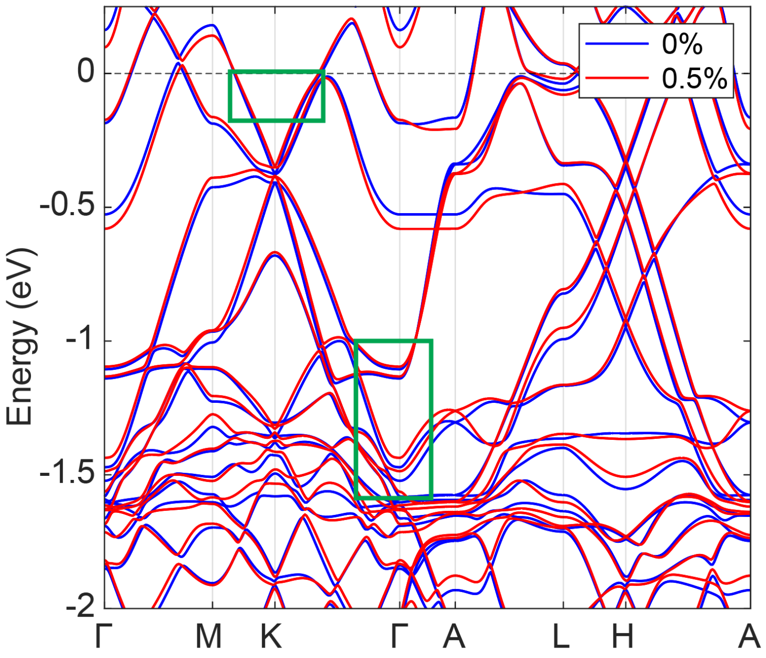

Supplementary Figure 6 DFT calculations as a function of strain. Red and blue bands are calculated with zero and 0.5% tensile strain, respectively.

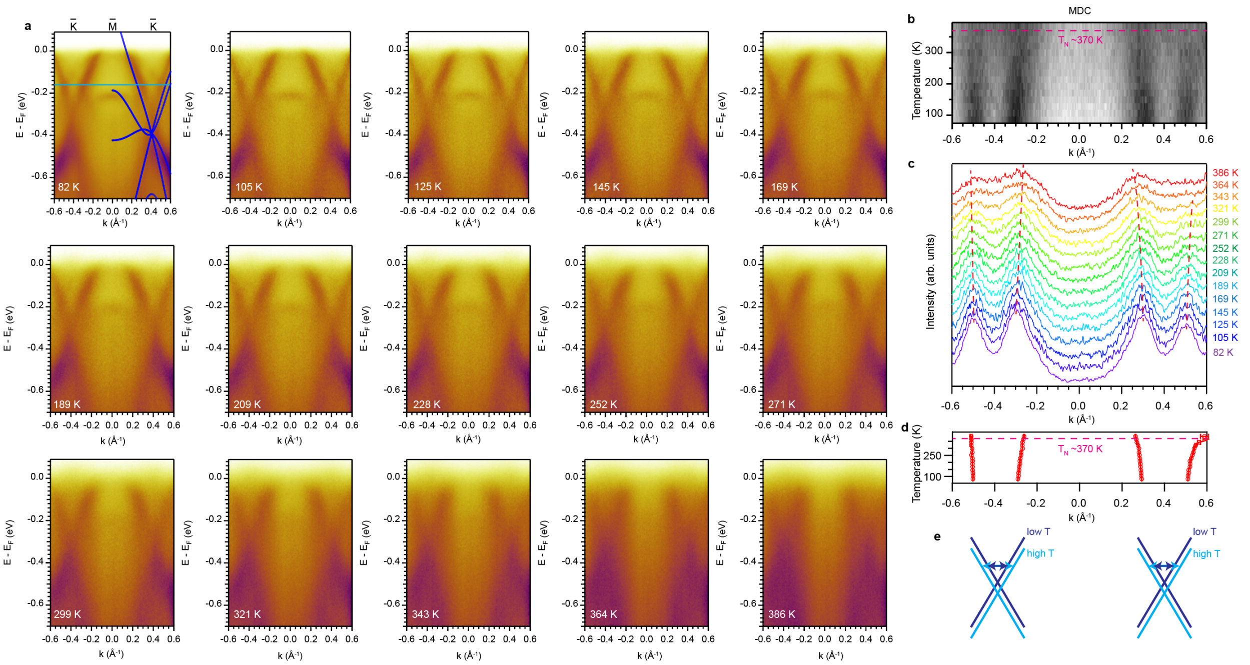

Supplementary Figure 7 Temperature evolution of the MDCs across the Dirac cones. a Raw data of the $\overline{\mathrm{K}}-\overline{\mathrm{M}}-\overline{\mathrm{K}}$ cut as a function of temperature. The two Dirac cones can be observed in the full temperature range from 82 K to 386 K. **b** MDCs along the green line in the first cut in **a**, plotted as a function of temperature to facilitate a better visualization of their temperature evolution. **c** Stack of MDCs similar to **b**, where four red dashed lines are guide to the eye for the peak shift. **d** Extracted peak positions as a function of temperature by fitting the MDCs with four Lorentzian functions and a constant background. Error bars are from the standard deviation resulting from the fitting process. **e** Schematic of the downward shift of the Dirac cone and the corresponding widening of the peak-to-peak spacing on warming.

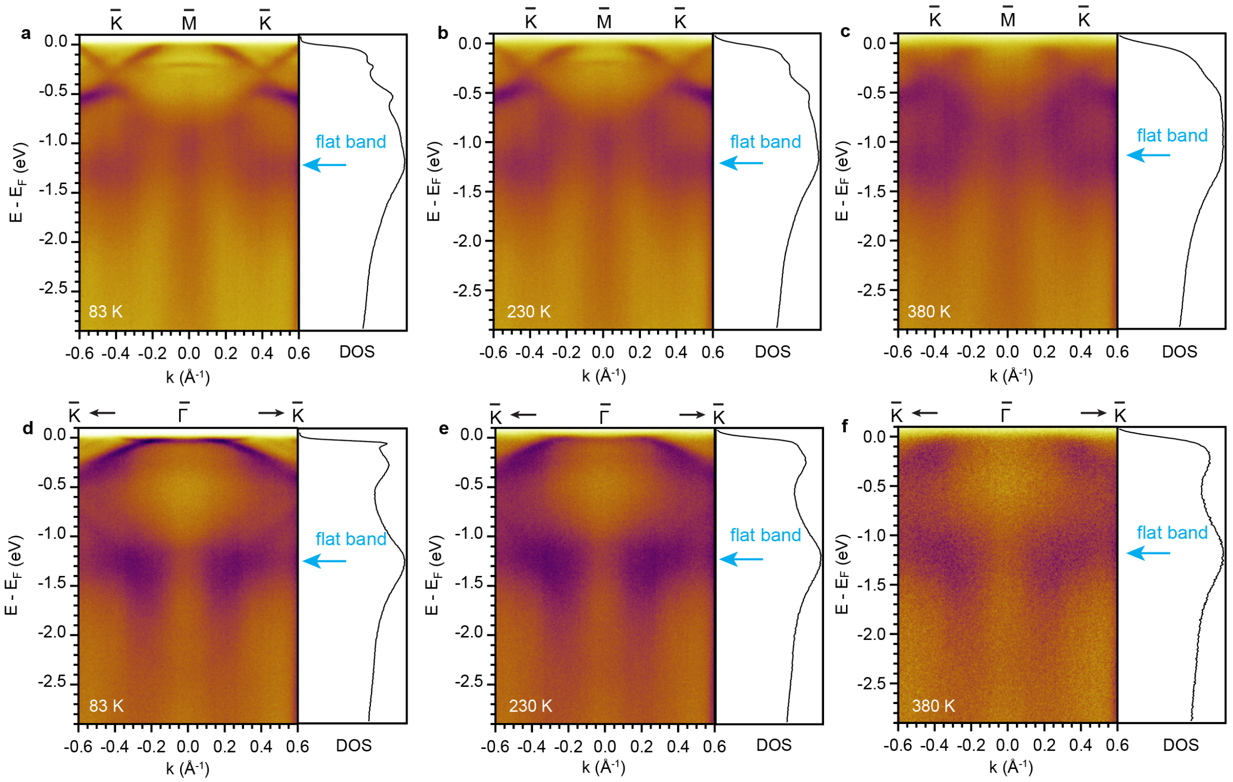

Supplementary Figure 8 Temperature dependence of the spin majority flat band. a-c $\overline{\mathrm{K}}-\overline{\mathrm{M}}-\overline{\mathrm{K}}$ cut taken at 83 K, 230 K and 380 K and their momentum-integrated EDCs, respectively. **d-f** $\overline{\mathrm{K}}-\overline{\Gamma}-\overline{\mathrm{K}}$ cut taken at 83 K, 230 K and 380 K and their momentum-integrated EDCs, respectively.

Supplementary Note 3 Photon-energy dependence measurement on FeSn thin film

Supplementary Figure 9 shows the photon-energy dependence of the $\bar{\Gamma}-\bar{\mathrm{K}}-\bar{\mathrm{M}}$ cut measured on the Se-capped and de-capped FeSn film. The out-of-plane constant energy contour (CEC) mapping at the binding energy of 1.15 eV displays a periodic pattern compatible with the Brillouin zone (BZ) of FeSn along the *c*-axis (*c*=4.46 Å), plotted with inner potential of 7 eV (Supplementary Figure 9 e). The helium-lamp photon energy 21.2 eV corresponds to the red arc in Supplementary Figure 9 e, which traverses from k_z=π/2 to k_z=π from the in-plane 1st BZ center to 2nd BZ center. In particular, k_z of the in-plane 2nd BZ center (k_x = 1.37 Å^{-1}), where the strongly renormalized electron-like bands are observed, is near k_z=π, justifying the comparison in Fig. 4a-c and g-i.

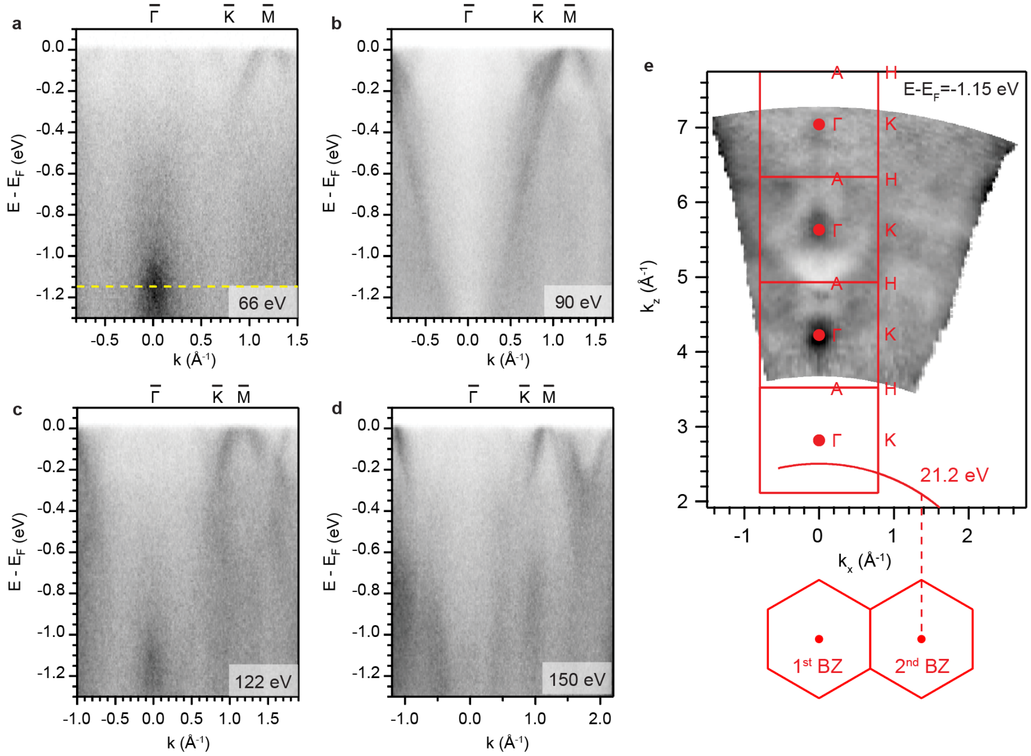

Supplementary Figure 9 Photon-energy dependence measurement on de-capped FeSn film. a-d $\bar{\Gamma}-\bar{K}-\bar{M}$ cut as a function of photon energy labeled on each cut. **e** Out-of-plane CEC mapping at -1.15 eV (yellow dashed line in **a**). BZ along the *c*-axis is overlaid on the ARPES map. Red arc corresponds to the helium-lamp photon energy of 21.2 eV.

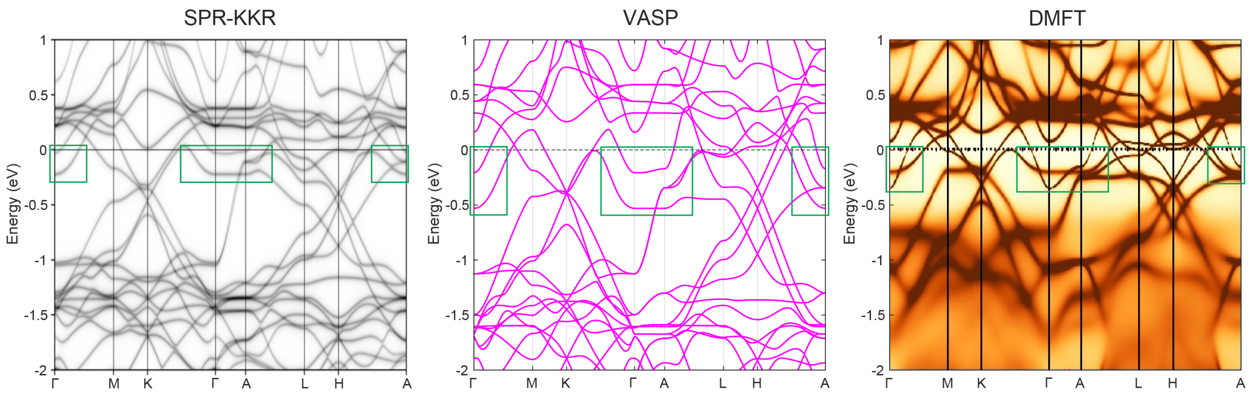

Supplementary Figure 10 Comparison between SPR-KKR, VASP and DMFT calculations. Green boxes mark the electron-like bands at Γ subject to the strongest renormalization effect.

Supplementary Note 4 DFT calculations of surface states

Supplementary Figure 11 shows the surface states on the Sn termination and the kagome termination calculated by DFT slab calculations. The kagome surface states deviate significantly from the ARPES results. The Sn surface calculation shows some resemblance with the bulk DFT calculation, but it does not exhibit the extremely narrow electron-like bands near E_F at Γ as seen in ARPES. Similar magnitude of renormalization is still required to adapt the Sn surface electron-like bands to the experimental ones. Therefore, one could not deem the surface states a more likely origin for the strongly renormalized bands than the bulk states.

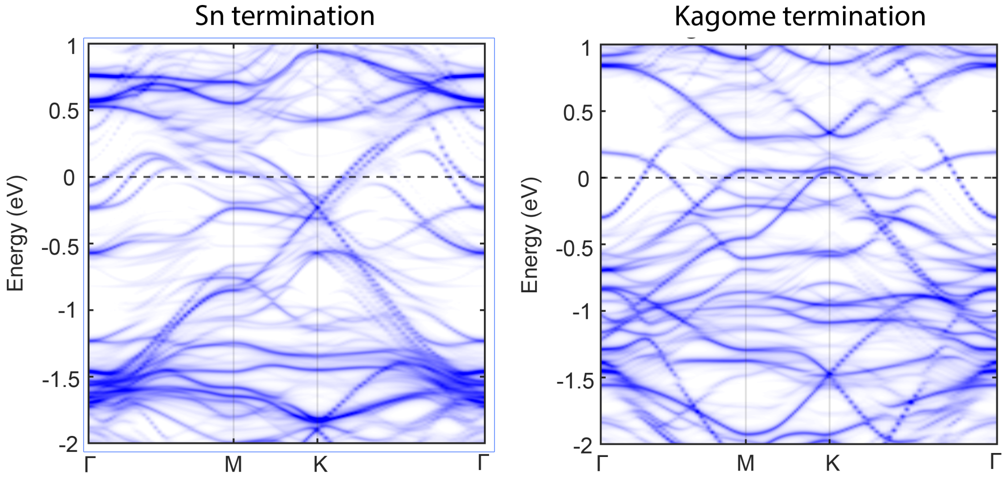

Supplementary Figure 11 Surface state DFT calculations for the Sn termination and the kagome termination.

Supplementary Note 5 Remarkable robustness of the strongly renormalized electron-like bands

it is extremely unlikely that the strongly renormalized bands are surface states because of their remarkable robustness against various perturbations. As we show in Supplementary Figure 12, these bands can survive thermal cycling from 81 K to 300 K back to 100 K, which is unexpected for typical surface states [6]. We also tried capping the as-grown FeSn films with amorphous Se and subsequently taking them to a synchrotron ARPES beamline and removing the capping layer by annealing. As shown in Supplementary Figure 12, while the synchrotron ARPES data quality on the samples that went through this brutal process is expectedly diminished compared to that taken directly after film growth, the strongly renormalized flower-shaped feature is still clearly observed. It is extremely unlikely that surface states can survive this process because of the additional deposition on the surface. In fact, slight deposition on a cleaved surface has been purposely employed to destroy the surface states and unravel the bulk states in ARPES experiments [7].

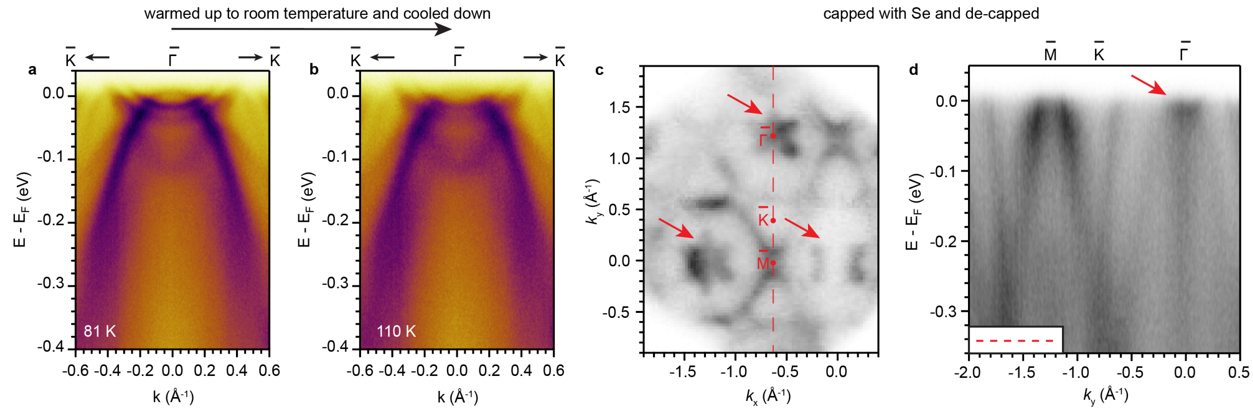

Supplementary Figure 12 Robustness of the strongly-renormalized bands. a,**b** $\overline{\mathrm{K}}-\overline{\Gamma}-\overline{\mathrm{K}}$ cut Before and after thermal cycling up to room temperature. **c**,**d** Fermi surface map and high symmetry cut after capping and de-capping the FeSn film with Se. Red arrows mark the persistent strongly-renormalized flower feature.

Supplementary Note 6 Termination-independence of the strongly renormalized electron-like bands

The strongly renormalized electron-like bands at Γ can be observed on both the kagome termination and the Sn termination, which directly rules out that these bands are surface states. We performed additional experiments using synchrotron ARPES with a 20-micron-sized beam spot, which enables resolving the two terminations. Because the FeSn films have a homogeneous Sn termination (Supplementary Figure 13), we cleaved FeSn single crystals and observed both terminations based on the distinct Sn core level profiles [8] (Supplementary Figure 14 b,e). The Fermi surface map on both terminations show an electron pocket at Γ, which has a very narrow bandwidth (Supplementary Figure 14 c,d,f,g). Although they do not exhibit the flower shape, the narrow bandwidth and large effective mass suggest a similar origin as the flower-shaped bands observed in the films. Importantly, this strongly renormalized electron-like band appears on both the Sn and the kagome terminations, indicating that it is not a surface state.

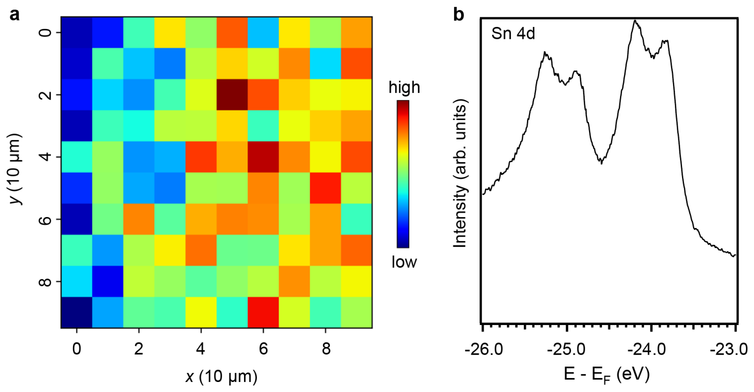

Supplementary Figure 13 Sn 4d core level spatial mapping on FeSn thin film. a spatial map of the overall intensity of the cut enclosing Sn 4d core levels in **b**. **b** Sn 4d core levels measured on the FeSn thin film sample.

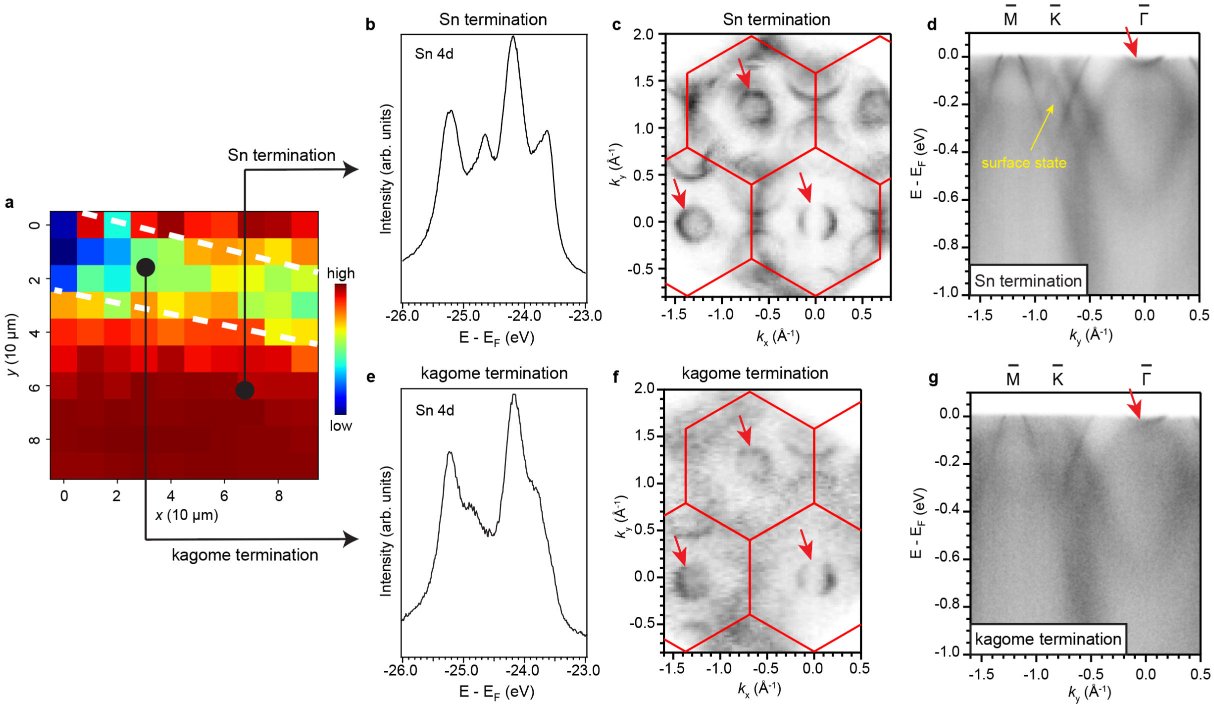

Supplementary Figure 14 Termination-independence of the renormalized electron-like bands. **a** spatial map of the overall intensity of the cut enclosing Sn 4d core levels as in **b** and **e**. Kagome termination is in between the two white dashed lines based on the core level profile and the lower intensity. The rest of the map is the Sn termination with higher intensity. **b**-**d** Sn termination core level, Fermi surface map and the $\bar{\Gamma} - \bar{K} - \bar{M}$ cut. **e**-**g** Data taken on the kagome termination corresponding to **b**-**d.** Photon energy used is 104 eV. Red arrows mark the renormalized electron-like bands at Γ. Yellow arrow marks the surface state on the Sn termination which is absent on the kagome termination.

Supplementary References

1. Bergman, D. L., Wu, C. & Balents, L. Band touching from real-space topology in frustrated hopping models. *Phys. Rev. B* **78**, 125104 (2008).

2. Soluyanov, A. A. & Vanderbilt, D. Wannier representation of Z_2 topological insulators. *Phys. Rev. B* **83**, 035108 (2011).

3. Chen, L. *et al.* Metallic quantum criticality enabled by flat bands in a kagome lattice. Preprint at https://arxiv.org/abs/2307.09431 (2023).

4. Hu, H. & Si, Q. Coupled topological flat and wide bands: Quasiparticle formation and destruction. *Sci. Adv.* **9**, eadg0028 (2023).

5. Sales, B. C. *et al.* Electronic, magnetic, and thermodynamic properties of the kagome layer compound FeSn. *Phys. Rev. Mater.* **3**, 114203 (2019).

6. Moore, R. G. *et al.* Topological electronic structure evolution with symmetry-breaking spin reorientation in $(Fe_{1-x}Co_x)$ Sn. *Phys. Rev. B* **106**, 115141 (2022).

7. Cheng, Z.-J. *et al.* Untangling charge-order dependent bulk states from surface effects in a topological kagome metal ScV_6Sn_6. *Phys. Rev. B* **109**, 075150 (2024).

8. Kang, M. *et al.* Dirac fermions and flat bands in the ideal kagome metal FeSn. *Nat. Mater.* **19**, 163 (2020).
